# Durable progression-free survival with first-line sintilimab plus chemotherapy followed by sintilimab maintenance in PD-L1-high recurrent cervical cancer: a case report

**DOI:** 10.3389/fonc.2026.1736364

**Published:** 2026-03-13

**Authors:** Juan Liu, Wang-Jing Ren

**Affiliations:** 1 Department of Oncology and Hematology, The People’s Hospital of Leshan, Leshan, Sichuan, China; 2 Department of Gynecology, The People’s Hospital of Leshan, Leshan, Sichuan, China

**Keywords:** complete response, immunotherapy, progression-free survival, recurrent advanced cervical cancer, sintilimab

## Abstract

**Background:**

Recurrent advanced cervical cancer has an extremely poor prognosis, with a 5-year survival rate of only 20%. Cervical cancer is closely linked to persistent infection with high-risk Human papillomavirus and its tumor microenvironment is often enriched with immune cell infiltrates, especially CD8+ T cells, indicating a degree of immunogenicity. Immunotherapy can prolong progression-free survival and overall survival in a subset of these patients, with the greatest benefit observed in those with programmed death-ligand 1positive tumors or squamous cell histology. Sintilimab is a fully human IgG4κ anti-PD-1 monoclonal antibody that has yielded positive results in lymphomas and several advanced solid tumors. However, published data on its use as a first-line therapy for recurrent advanced cervical cancer remain scarce.

**Case presentation:**

We report a case involving a 51-year-old Chinese woman whose advanced cervical cancer recurred with bilateral lung metastases 3 years after radical surgery and radiotherapy. First-line treatment with sintilimab plus chemotherapy, followed by maintenance therapy with sintilimab, achieved a complete response and progression-free survival of 37.5 months. Only grade 2 or lower immune-related adverse events occurred: grade 1 subclinical hyperthyroidism and grade 2 leukopenia.

**Conclusions:**

Maintenance therapy with sintilimab after first-line treatment with sintilimab plus chemotherapy can confer substantial clinical benefits in patients with recurrent advanced cervical cancer, with a favorable safety profile and no serious adverse events observed, warranting further prospective investigation.

## Introduction

Cervical cancer is the fourth most frequently diagnosed malignancy and the fourth leading cause of cancer-related death among women worldwide (1). In 2022, approximately 660,000 new cases of cervical cancer were diagnosed worldwide, with 350,000 deaths (2). In China, approximately 150,700 new cases and 55,700 related deaths have been reported (3). Recurrent advanced cervical cancer has an extremely poor prognosis, with a 5-year survival rate of only 20%. The current National Comprehensive Cancer Network (NCCN) guidelines recommend chemotherapy plus pembrolizumab (KEYNOTE-826) or atezolizumab (BEATcc phase III study), with or without bevacizumab, as standard first-line treatment for programmed death-ligand 1 (PD-L1)positive (combined positive score [CPS] ≥ 1) recurrent or metastatic advanced cervical cancer. With this regimen, the median progression-free survival(PFS) is 10–13 months, and the median overall survival (OS) is 26–36 months (4–6).

KEYNOTE-826 included only 16 patients from Taiwan, China, but did not include patients from mainland China. The BEATcc trial also did not include any Chinese population. This failure to include a Chinese population created a gap in research on first-line immunotherapy with PD-1/PD-L1 inhibitors in Chinese patients with cervical cancer. As a fully humanized IgG4 monoclonal antibody against PD-1, sintilimab restores T-cell-mediated antitumor immunity by blocking PD-1/PD-L1 interaction, which is consistent with the mechanism of pembrolizumab described in KEYNOTE-826. Treatment with sintilimab has yielded positive results in patients with lymphomas and multiple solid tumors. However, published reports on the use of sintilimab as a first-line therapy for recurrent advanced cervical cancer are very limited. Here, we report a patient with recurrent cervical cancer presenting as bilateral pulmonary metastases after prior radical surgery and radiotherapy, who achieved complete remission and durable progression-free survival of 37.5 months on first-line sintilimab plus chemotherapy followed by maintenance sintilimab.

## Case description

The patient was a 51-year-old Chinese woman who had undergone bilateral tubal ligation more than 20 years earlier and had received benzathine penicillin for latent syphilis. She also had a history of paroxysmal supraventricular tachycardia (PSVT), which occurred occasionally; she declined catheter ablation for PSVT. On December 10, 2019, bilateral ureteral stents were inserted because of hydronephrosis. She was a never-smoker, did not drink alcohol, and never experienced abnormal vaginal bleeding. Her father was deceased; her mother, who had hypertension, was still alive. She has two healthy daughters.

**Figure 1:**
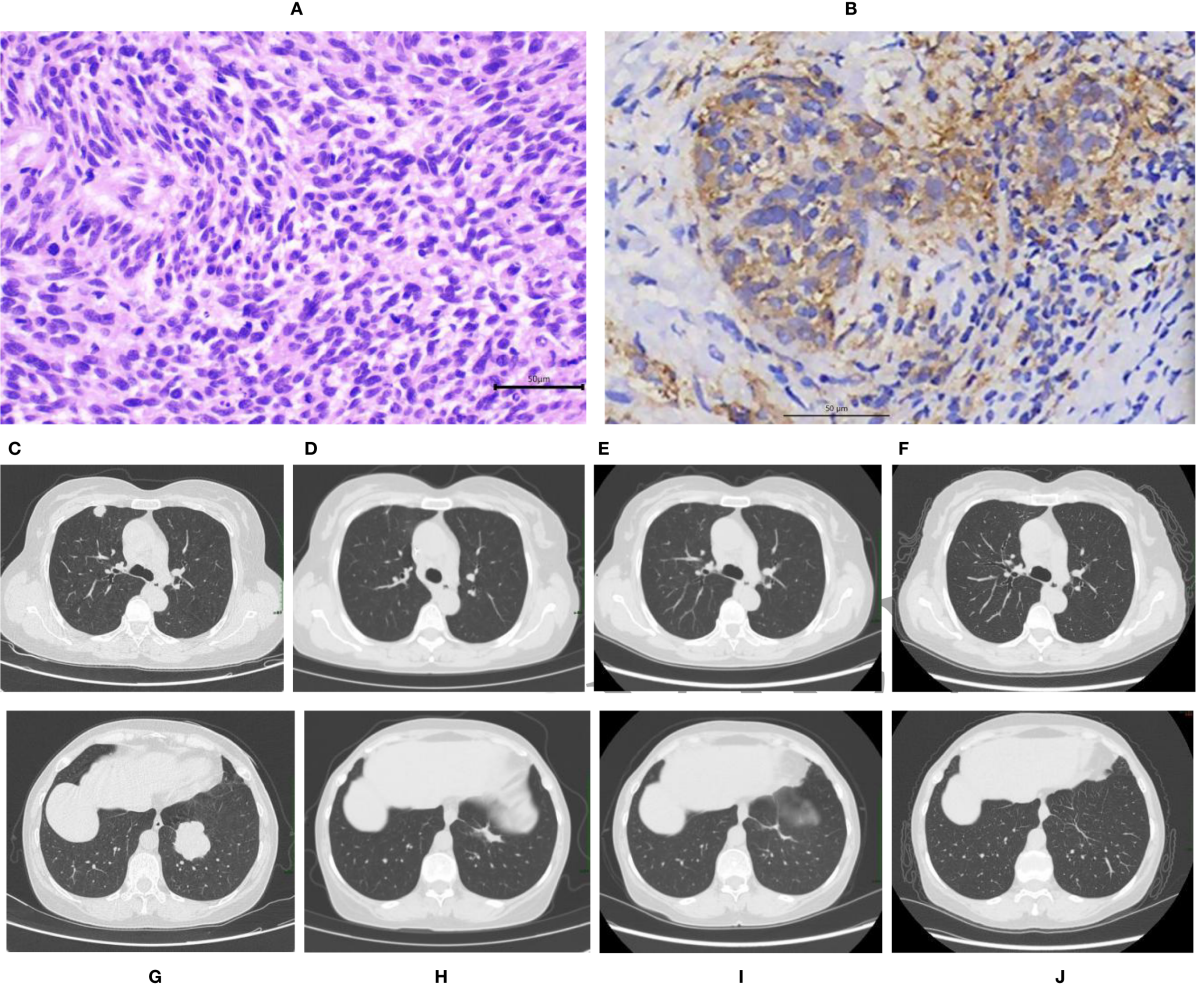
**(A–J)** Pathological findings and CT imaging results. Pathological findings **(A, B)**. Poorly differentiated squamous cell carcinoma of the cervix, as demonstrated by hematoxylin and eosin (H&E) staining. The magnification ratio is 400× **(A)**. Immunohistochemistry revealed PD-L1(+) (CPS 60), which was detected using an anti-PD-L1(22C3) antibody with a DAKO Autostainer Link48.The magnification ratio is 200× **(B)**.CT images **(C–J)**. At recurrence, metastases were present in the right upper lobe(1.6x1.2cm) and left lower lobe(4.2x3.8cm) of the lung **(C, G)**. After 6 cycles of sintilimab plus chemotherapy, her bilateral pulmonary metastases markedly decreased(right upper lobe 0.8x0.4cm, left lower lobe 2.6x0.8 cm) **(D, H)**. The bilateral pulmonary metastases continued to shrink after 24 months of therapy (right upper lobe 0.7x0.2 cm, left lower lobe 1.9x0.5 cm) **(E, I)**. A CR of the bilateral pulmonary metastases was achieved after 37.5 months of therapy (fibrotic streaking only) **(F, J)**.

On October 14, 2019, Human papillomavirus (HPV) testing was performed using Hybrid Capture 2 (HC2), which was positive for high-risk HPV types. The RLU/CO was 24.96. However, specific genotyping was not conducted at that time. On October 17, 2019, a routine physical examination revealed a solid cervical mass. Cervical biopsy confirmed the presence of cervical cancer. On October 22, 2019, surgical treatment was performed via laparotomy under general anesthesia. The procedure included type C2 radical hysterectomy + pelvic lymphadenectomy + para-aortic lymph node sampling. Pathology revealed a 4 × 2 × 1.5 cm poorly differentiated squamous cell carcinoma involving the full thickness of the cervical wall (**Figure 1A**). No lymphovascular or perineural invasion was present, and all 19 lymph nodes were reactive. On the basis of the 2018 International Federation of Gynecology and Obstetrics (FIGO) staging system, the patient had FIGO stage IB2 disease. Pelvic external beam radiotherapy (EBRT) was delivered starting on December 23, 2019 (the target volume PTV was 50 Gy in 25 fractions of 2 Gy). The patient refused concurrent platinum-containing chemotherapy and brachytherapy (BT). The patient did not undergo regular follow-up examinations after the completion of treatment.

In November 2022, three years after her primary treatment, the patient developed dry cough and progressive, left-sided chest pain that was unrelated to deep breathing or posture. The symptoms were initially attributed to a “common cold” by her primary care physician; however, after 2 weeks of nonresponse to empirical antibiotics and antitussives, she returned for further evaluation. On November 24, 2022, her serum squamous cell carcinoma antigen (SCC) concentration was 49.40 ng/mL, and chest computed tomography (CT) revealed new metastases in the left lower and right upper lobes. Physical examination revealed no palpable peripheral lymphadenopathy, clear lungs without crackles, regular heart rhythm with normal heart sounds and no murmurs, a soft abdomen with old surgical scars but no tenderness or rebound, no organomegaly, negative shifting dullness, normal bowel sounds, and no lower-limb edema. The pulmonary mass was deeply located, posing a high risk for percutaneous biopsy. Owing to limited financial resources, considerable concerns regarding the risks of pneumothorax and hemorrhage associated with the procedure, and the urgent need to initiate treatment, the patient provided written informed refusal of lung biopsy for pathological confirmation. Following discussion with two radiologists (who independently reviewed the imaging results), we reached a consensus that the enhancement pattern on contrast-enhanced CT suggested a high probability of malignancy. The markedly elevated SCC-Ag level indicated the presence of squamous cell carcinoma. This patient was a never-smoker, and imaging revealed multiple round-to-oval masses in the right upper and left lower lobes with shallow lobulation but without spiculation, pleural retraction, or hilar/mediastinal lymph node involvement. We respected the patient’s refusal of repeat biopsy. Considering her history of prior cervical cancer, elevated SCC levels, and radiological findings, the diagnosis of pulmonary metastasis from cervical cancer was favorable. (**Figures 1C, G**). The patient had FIGO stage IVB disease. Programmed death-ligand 1testing was positive (CPS 60, 22C3 antibody) (**Figure 1B**). Owing to the high costs, she refused pembrolizumab and bevacizumab and opted for off-label treatment with sintilimab. Starting on December 13, 2022, she received first-line immunotherapy plus albumin-bound paclitaxel and cisplatin (TP) chemotherapy for six 3-week cycles: sintilimab (200 mg) on day 1, albumin-bound paclitaxel (300 mg) on day 2, and cisplatin (40 mg) on days 2–3. Her serum SCC level normalized after cycle 2 and has remained within the normal range since then. Two radiologists independently reviewed the images without knowledge of each other’s interpretations. After 4 cycles, chest CT revealed marked shrinkage of the lung metastases; after 6 cycles, the tumors continued to regress (**Figures 1D, H**). The response was evaluated as a partial response (PR) according to the Response Evaluation Criteria in Solid Tumors version 1. 1(RECIST v1. 1). After she completed all six cycles, 200 mg of sintilimab was given every 3 weeks for maintenance therapy.

**Figure 2:**
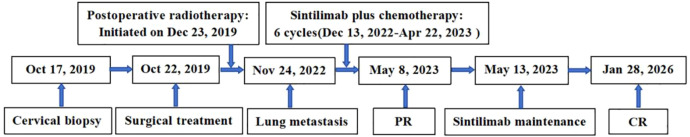
Timeline of patient’s treatment.

A CT scan after 24 months of treatment revealed continued shrinkage of the bilateral pulmonary metastases (**Figures 1E, I**). Hence, we suggested stopping immunotherapy, but the patient, who experienced no significant immune-related adverse events, was extremely anxious about a relapse and insisted on continuing. After full discussion, her wishes were respected, and the treatment interval was extended to every 4 weeks. On January 28, 2026, the bilateral pulmonary metastases exhibited a complete response (CR) after 37.5 months of therapy (**Figures 1F, J**). RECIST v1. 1 was used to evaluate treatment response. PFS was defined as the time from the start of the first cycle of immunotherapy plus chemotherapy to tumor progression or death. The timeline of the patient’s treatment is shown in **Figure 2**.

Throughout treatment, the observed treatment-related adverse reactions were as follows: grade II myelosuppression, which occurred during chemotherapy and resolved after granulocyte-stimulating therapy; grade I subclinical hyperthyroidism, which occurred during maintenance immunotherapy and did not require treatment; grade 2 leukopenia, which was treated by withholding sintilimab and administering granulocyte-stimulating agents, after which immunotherapy was resumed; and grade 2 anemia, which was managed supportively. The changes in blood counts, SCC, and thyroid-stimulating hormone (TSH) levels are summarized in **Table 1**.

**Table 1:** Changes in blood count, SCC, and TSH levels.

TIME	SCC (0-2.7 ng/mL)	WBC (3.5-9.5x109/L)	NEUT (1.8–6.3x109/L)	Hb (115–150 g/L)	PLT (125-3 50x109/L)	TSH (0.55–4.78mIU/L)
Initial treatment	49.40	3.18	2.57	126	240	0.771
Two cycles	0.95	2.37	1.80	106	194	0.690
Four cycles	0.63	4.64	3.84	108	128	0.755
Six cycles	0.92	3.28	2.58	109	164	0.113
12 months	0.85	2.00	1.48	94	154	1.249
24 months	0.71	2.50	1.95	104	105	0.509
37.5 months	1.06	2.47	1.88	112	156	0.63

## Discussion

This patient presented with advanced, recurrent disease accompanied by bilateral pulmonary metastases three years after radical surgery and radiotherapy. PD-L1 testing was positive (CPS 60). She received TP chemotherapy plus sintilimab followed by maintenance therapy with sintilimab, achieving CR and a PFS of 37.5 months, without grade 3 or higher adverse events. This patient did not receive bevacizumab, making the regimen economically feasible. Throughout the treatment, the patient’s preferences and financial situation were fully considered, and both efficacy and patient satisfaction were high.

The phase III GOG-240 trial revealed that adding bevacizumab to chemotherapy prolonged overall survival (17.0 vs. 13.3 months) (7, 8). Although targeted therapies have improved treatment outcomes, the benefit remains limited. Persistent HPV infection with high-risk types 16 and 18 drives cervical carcinogenesis. Human papillomavirus early proteins 6/7 (HPV E6/E7) activate the PI3K–Akt, STAT3 and NF-κB pathways, thereby upregulating PD-L1 expression, inducing indoleamine 2,3-dioxygenase 1 (IDO1) activity, and downregulating human leukocyte antigen class I (HLA-I) antigen presentation (9, 10).

Numerous studies have revealed that the expression of PD-L1 is related to the treatment and prognosis of cervical cancer. Moreover, the rate of PD-L1 positivity in patients with cervical cancer ranges from 35-96%,with squamous cell carcinoma expressing PD-L1 more frequently than adenocarcinoma does, providing a biological rationale for immune checkpoint blockade (11). PD-1 inhibitors exert their efficacy through two main mechanisms. First, direct blockade prevents PD-1 from binding to its ligands PD-L1 and PD-L2, thereby restoring T-cell activation. Second, indirect effects include restoration of IFN-γ signaling, which subsequently downregulates IDO1 activity and upregulates HLA-I antigen presentation, ultimately reactivating suppressed CD8+ T cells. The efficacy of immunotherapy in cervical cancer is associated not only with PD-L1 expression, but also with tumor microenvironment (TME) status. Based on the number and localization of CD8+ T cells, the TME is classified into three subtypes: inflamed (34%), excluded (56%), and cold (10%) (12).Cervical cancer exhibits a “decoupling” phenomenon between PD-L1 expression and TME classification. High PD-L1 expression may be detected in all subtypes, but only the inflamed (hot) type demonstrates IFN-γ-mediated adaptive induction with functional T cell infiltration, conferring sensitivity to PD-1 inhibitors. Excluded-type tumors, where T cells are physically excluded from tumor epithelium despite stromal infiltration, and cold-type tumors, which lack T cell infiltration but harbor intrinsic oncogenic PD-L1 expression, both demonstrate limited efficacy with PD-1 inhibitor monotherapy. However, they require distinct combination strategies: excluded types necessitate barrier-disrupting approaches (chemotherapy/radiation), while cold types benefit from immune-activating regimens or myeloid derived suppressor cells (MDSC) depletion (13).

KEYNOTE-826 and BEATcc have established immune checkpoint inhibitors plus chemotherapy as standard first-line therapy for PD-L1-positive recurrent/metastatic cervical cancer. Even though bevacizumab was mandatory in BEATcc and used in 63% of patients included in KEYNOTE-826, we observed durable complete remission without bevacizumab in our patient, suggesting that selected patients with high PD-L1 expression may achieve exceptional long-term outcomes with chemoimmunotherapy alone while avoiding antiangiogenic toxicity (4–6). Data on sintilimab in this setting remain scarce. This patient’s 37.5-month PFS without bevacizumab is a real-world observation of durable remission with chemoimmunotherapy alone, although validation in controlled studies is needed. We acknowledge that this single case cannot establish the noninferiority of bevacizumab omission.

We attribute this patient’s complete response (CR) and 37.5-month progression-free survival (PFS) to her high PD-L1 expression (CPS 60), suggesting that high PD-L1 expression may identify a “super-responder” subgroup. We emphasize that this outcome may not be applicable to patients with PD-L1-negative or low-expression tumors, underscoring the importance of biomarker-driven patient selection in future clinical practice. The real-world study by Zhou et al. (2025) confirms the efficacy of sintilimab as first-line therapy for recurrent cervical cancer and demonstrates that earlier initiation of sintilimab is associated with better outcomes (14). This aligns with our case: our patient received sintilimab immediately upon recurrence, completed six cycles of combination therapy, and was then maintained on sintilimab for more than two years—factors that may contribute to sustained efficacy but require prospective validation. We acknowledge the limitations of this observation. We clearly state that this single-case report is hypothesis-generating and cannot establish causality or generalizability. Prospective studies with larger cohorts are essential.

A phase II study of sintilimab plus albumin-bound paclitaxel as second-line therapy for PD-L1-positive recurrent cervical cancer reported an Objective Response Rate (ORR) of 44% and a median PFS of 5.2 months (15). This case observation suggests extending the use of sintilimab to the first-line setting, though prospective trials are needed to establish efficacy in this population.Sintilimab has shown high efficacy as a first-line treatment for nonsquamous (ORIENT-11) and squamous (ORIENT-12) NSCLC as well as for esophageal squamous carcinoma (ORIENT-15), supporting its broad immunomodulatory profile (16–18). Whether similarly intensified regimens could improve responses in patients with cervical cancer merits prospective investigation. The patient has now completed 37.5 months of immunotherapy. At year 2, we suggested discontinuation; however, the patient, who was free of significant irAEs, strongly wished to continue treatment due to fear of relapse. After counseling, we extended the dosing interval to every 4 weeks. CheckMate-153 in patients with NSCLC showed that continuous nivolumab resulted in longer median PFS compared with 1-year fixed-duration therapy (24.7 vs. 9.4 months) (19); this benefit was maintained in patients with CR/PR at randomization (31.0 vs. 10.6 months), providing a rationale for our cautious extension of sintilimab while balancing cumulative toxicity and patient preference. The results presented in this case may provide preliminary insights, if validated in prospective cohorts. At 37.5 months, the patient achieved CR according to RECIST v1.1. We recommended either discontinuing therapy or extending the treatment interval to every 6 weeks while continuing long-term surveillance. Admittedly, this remains an exploratory approach. Regrettably, her circulating tumor DNA (ctDNA) was not assessed at relapse or during follow-up because of cost, preventing us from exploring early molecular clearance as a surrogate endpoint.

## Conclusion

In summary, first-line chemotherapy plus sintilimab followed by maintenance therapy with sintilimab proved to be exceptionally effective, with an acceptable safety profile in this patient with recurrent advanced cervical cancer. The limitations include the absence of biopsy confirmation of lung metastases, the single-patient design, the short follow-up period, the lack of bevacizumab, and the absence of ctDNA data. Should these preliminary observations prove reproducible in larger cohorts, prospective studies might first explore the potential role of sintilimab in first-line cervical cancer treatment; subsequently, comparative trials could investigate the optimal duration of therapy, such as fixed-duration versus extended regimens.

## Patient perspective

When I was told that the cervical cancer had come back and spread to both lungs, I felt hopeless; the only treatments the doctors offered first were very expensive imported drugs that my family could not afford. I was extremely worried that I would have to stop treatment because of cost. Fortunately, my oncologist explained that an alternative PD-1 inhibitor called sintilimab was available at a much lower price and that I could receive it together with chemotherapy. After six cycles, my dry cough and chest pain disappeared, and follow-up CT scans revealed that the lung nodules had shrunk. I experienced only mild fatigue and temporary low white cell counts; I could still cook, walk in the park, and take care of my grandchildren. The most difficult part was anxiety during the first year, fearing every new scan, but when the doctor said the tumors had completely disappeared, I cried with relief. I have been on sintilimab maintenance therapy for more than three years with no serious side effects. I am thankful that an affordable immunotherapy has allowed me to live a normal life again, and I hope this case encourages other patients who cannot afford expensive medicine.

## Data Availability

The original contributions presented in the study are included in the article/supplementary material. Further inquiries can be directed to the corresponding author.

## Glossary

BT: brachytherapy
CD8+ T cells: cluster of differentiation 8 positive T cells
CR: complete respon, CT, computed tomography
ctDNA: circulating tumor DNA
CPS: Combined positive score
EBRT: Pelvic External Beam Radiotherapy
FIGO: International Federation of Gynecology and Obstetrics
Hb: hemoglobin
HC2: Hybrid Capture
E: hematoxylin and eosin
HPV E6/7: Human papillomavirus early protein 6/7
HLA-I: Human leukocyte antigen class I
IDO1: Indoleamine 2,3-dioxygenase 1
IFN-γ: Interferon-gamma
irAEs: immune-related adverse events
NCCN: National Comprehensive Cancer Network
NEUT: Neutrophil granulocyte
NF-κB: Nuclear factor kappa-light-chain-enhancer of activated B cells
NSCLC: Non-small cell lung cancer
OS: Overall survival
ORR: Objective response rate
PFS: Progression-free survival
PSVT: Paroxysmal supraventricular tachycardia, PTV, Planning target volume
PD-1: Programmed death 1
PLT: Platelet
PI3K–Akt: Phosphoinositide 3 kinase–protein kinase B
RECIST v 1. 1: Response Evaluation Criteria in Solid Tumors version 1. 1
SCC: squamous cell carcinoma antigen
TSH: Thyroid-stimulating hormone
TP: Albumin-bound paclitaxel and cisplatin
WBC: White blood cell
STAT3: Signal transducer and activator of transcription 3.
